# The effect of prolonged complex decongestive therapy for lower limb secondary lymphedema

**DOI:** 10.1016/j.jvsv.2026.102444

**Published:** 2026-01-07

**Authors:** Kotaro Suehiro, Takasuke Harada, Yuriko Takeuchi, Takahiro Mizoguchi, Hiroshi Kurazumi, Mototsugu Shimokawa, Kimikazu Hamano

**Affiliations:** a Department of Surgery and Clinical Science, Yamaguchi University Graduate School of Medicine, Ube, Yamaguchi, Japan; b Department of Biostatistics, Yamaguchi University Graduate School of Medicine, Ube, Yamaguchi, Japan

**Keywords:** Leg, Lymphedema, Complex decongestive therapy, Adherence, Ageing

## Abstract

**Objective:**

To clarify the effect of prolonged complex decongestive therapy (CDT) on limb volume in patients with lower limb lymphedema (LLL).

**Methods:**

We reviewed patients who first visited our clinic for cancer-related LLL between April 2009 and March 2015 and continued CDT at our clinic for 7 years or longer. At 6- to 12-month intervals, limb volume was calculated from tape measurements, and ultrasound examination was performed to evaluate changes in the skin and subcutaneous tissue conditions.

**Results:**

In 52 patients (68 lower limbs) who were on CDT for a median of 11.2 years, 54% maintained reduced limb volume, whereas limb volume increased in 46% of patients compared with their initial visits. An increase in circumference was mainly observed in the calf area, where the subcutaneous echo-free space (edema) increased. In multivariate analysis, relevant factors associated with the increase in limb volume were an increase in body mass index (odds ratio, 1.73; 95% confidence interval [CI], 1.15-2.93), the use of compression devices for fewer than 5 days per week (odds ratio, 4.57; 95% CI, 1.06-23.53), and the use of compression devices with interface pressure lower than 20 mm Hg (odds ratio, 36.47; 95% CI, 3.42-1061.90).

**Conclusions:**

During prolonged CDT, limb volume increased in 46% of limbs with LLL, which was associated with an increase in edema, particularly in the calf area. The factors associated with increased limb volume were an increase in body mass index, the use of compression devices for fewer than 5 days per week, and the use of compression devices with interface pressure <20 mm Hg.

**Article Highlights**

• **Type of Research:** Single-center, retrospective cohort study
• **Key Findings:** During a median of 11.2 years of complex decongestive therapy for lower limb lymphedema (68 limbs), the limb volume increased in 31 limbs (46%). The significant factors associated with an increase in limb volume were increased body mass index and poor adherence to compression therapy.
• **Take Home Message:** Maintaining a stable body mass index and adherence to compression therapy are critical for maintaining reduced limb volume in prolonged complex decongestive therapy.

Currently, complex decongestive therapy (CDT) is the primary treatment for peripheral lymphedema. The first phase of CDT consists of skin care, manual lymphatic drainage, exercise, and compression therapy, typically using bandages; however, this is replaced by elastic stockings, sleeves, or other types of compression devices in the second phase.^1^ To date, the efficacy of CDT has been reported mostly in upper limb lymphedema, whereas reports regarding CDT for lower limb lymphedema (LLL) are scarce. Furthermore, the observation periods in these studies were not long.^2^, ^3^, ^4^, ^5^ We previously reported the results of CDT for LLL with a median treatment duration of 3 years.^6^ In that study, we found that the limb volume after CDT was larger than that at the initial visit in 35% of patients, and the causes of these increases were exacerbated edema and possible adipose tissue proliferation, particularly in the lower medial thigh.

Because lymphedema is a noncurable disease, CDT must be continued lifelong. When CDT is provided for a prolonged period, the effect of the first phase is inevitably diluted, and the quality of the second phase becomes more critical. As patients age during years of CDT, additional factors related to aging emerge, which may worsen edema and reduce adherence to CDT. Therefore, it is important to know what can or cannot be achieved through prolonged CDT to manage patients properly over a long period. In this study, we reviewed patients with cancer-related LLL who received CDT at our clinic for a prolonged period and studied the changes in limb volume, circumference, and skin and subcutaneous tissue conditions. Based on these observations, we discussed the factors particularly related to the increase in leg volume during prolonged CDT.

## Methods

This retrospective study was approved by the institutional review board of Yamaguchi University Hospital (Ube, Yamaguchi, Japan; approval number 2025-027), and the need for individual patient consent was waived. Between April 2009 and March 2015, a total of 127 patients with cancer-related LLL visited our clinic. Of those, the patients who were treated in our clinic for 7 years or longer were included in this study. Patients with known systemic edemagenic conditions, such as cardiac, hepatic, or renal failure, or end-stage cancer at their initial visit, were excluded. Patients complicated with chronic venous insufficiency were also excluded because they were often complicated by dermatitis and ulcers, each of which causes inflammatory swelling (edema) and requires management different from standard CDT. Patients who underwent reductive surgery, that is, liposuction and Charles procedure, were excluded to evaluate the sole effect of CDT on limb volume. The remaining 52 patients were included in the study. Although the factors related to cancer treatment, that is, surgery, radiation, metastases, chemotherapy, and so on, could affect the outcome of CDT, these could not be included in this study because approximately half of the patients were referred from other hospitals and detailed information could not be obtained in these patients.

CDT was provided as advocated by the International Society of Lymphology.^1^ The clinical stage of lymphedema was determined based on the Consensus Document of the International Society of Lymphology.^1^ At 6- to 12-month intervals, limb volume was calculated from tape measurements using the truncated cone method,^7^ and skin and subcutaneous tissue ultrasound examination was performed. The skin and subcutaneous tissues were scanned using an ultrasound system (LOGIQ S6; GE Healthcare, or Xario 200G; Canon Medical Systems Corporation) with an 8-12 MHz linear transducer at eight anatomical points (upper, lower, medial, and lateral points on the thigh and leg). Subcutaneous echo-free space (SEFS), which represents the local edema severity, was graded as follows: grade 0, no SEFS; grade 1, horizontally oriented (<45° to the skin) SEFS only; grade 2, vertically oriented (≥45° to the skin) SEFS bridging the horizontally oriented SEFSs, as we previously reported.^8^ The excellent inter- and intrarater reliability of this grading system was demonstrated by Giray et al.^9^ Ultrasound images were acquired by a single qualified technician and were graded by a single interpreter (K.S.).

### Statistical analysis

Results are expressed as median (range) or count unless otherwise indicated. The Wilcoxon signed-rank test was used to evaluate changes in limb volume, circumference, and ultrasonographic measurements over time. The Mann-Whitney *U* test was used to evaluate the differences between groups. The χ^2^ test was used to assess the categorical variables, including SEFS grades, adherence to compression therapy, and compression levels. The changes in SEFS grades are presented as mean values because they appear blurred when expressed as median values. The linear regression analysis was used to test the correlation between the change in body mass index (BMI) and the change in leg volume. To express the changes in measurements before and after CDT, the ratios of the changes were calculated as follows:

Ratio of the change in measurements (%) = ([measurement in the last visit – measurement in the initial visit]/measurement in the initial visit) × 100.

Logistic regression analyses were performed to identify the factors that significantly affected the increase in limb volume. Statistical analyses were performed using JMP 11.0 (SAS Institute). A *P* value <.05 was considered statistically significant.

## Results

### Changes in patient characteristics and lower limb conditions

The patient characteristics and lower limb conditions during the initial and latest visits are summarized in Table I. The follow-up period was 11.2 (7.0-15.4) years. During this period, the median patient age advanced from 65 to 75 years, and the rate of patients aged 75 years or older increased from 22% to 50% (*P* < .01). In three patients with unilateral LLL (9%), the contralateral leg developed lymphedema symptoms, resulting in bilateral LLL. In five legs (7%), which were all stage II, lymphedema symptoms disappeared; namely, they became stage 0.

**Table I:** Patient characteristics and lower limb conditions

Patient characteristics	Initial visit	Latest visit
	All patients, n = 52; group A, n = 28; group B, n = 24	
Age, years	65 (35-84)	75 (50-95)^a^
Group A	66 (45-84)	76 (54-95)^a^
Group B	64 (35-84)	74 (50-93)^a^
Sex (female:male)	48:4	No change
Group A	25:2	
Group B	23:2	
Body mass index, kg/m^2^	24 (17-34)	23 (18-34)
Group A	24 (17-34)	24 (18-34)
Group B	23 (17-29)	23 (18-29)
Laterality		
Unilateral (right:left)	36 (17:20)	33 (15:18)
Group A	18 (8:10)	18 (8:10)
Group B	18 (10:8)	15 (9:6)
Bilateral	16	19
Group A	10	10
Group B	6	9
CDT period, years	0	11.2 (7.0-15.4)
Group A	0	10.5 (7.0-15.3)
Group B	0	12.1 (7.0-15.4)

Leg conditions	All legs, n = 65; group A, n = 37; group B, n = 28	All legs, n = 68; group A, n = 37; group B, n = 31
International Society of Lymphology stage		
0	0	5
Group A	0	5
Group B	0	0
II	60	58
Group A	35	30
Group B	25	28
III	5	5
Group A	2	2
Group B	3	3
Volume, mL	7218 (5090-12,690)	7309 (4621-12,137)
Group A	7522 (5297-12,690)	6262 (4621-11,553)^a^
Group B	7126 (5090-10,890)	8245 (6261-12,137)^a^
Circumferences, cm		
20 cm above the patella	53.7 (41.4-78.6)	53.4 (40.3-68.5)
Group A	55.4 (45.8-78.6)	52.8 (40.3-68.5)^a^
Group B	52.0 (41.4-57.9)	54.4 (44.3-62.5)^a^
Upper edge of the patella	40.6 (31.6-58.0)	40.7 (31.3-53.8)
Group A	41.2 (33.7-58.0)	38.2 (31.3-53.8)^a^
Group B	39.3 (31.6-47.1)	43.6 (38.5-50.3)^a^
30 cm below the patella	24.7 (19.8-41.5)	25.1 (16.3-43.2)
Group A	24.3 (20.1-34.5)	23.0 (16.3-35.5)^a^
Group B	25.3 (19.8-41.5)	27.7 (21.8-43.2)^a^
Foot	22.6 (18.5-27.3)	22.0 (18.4-28.2)^a^
Group A	22.6 (20.5-26.2)	21.6 (19.8-25.6)^a^
Group B	23.0 (18.5-27.3)	22.7 (18.4-28.2)

Group A: legs that maintained decreased volume during complex decongestive therapy.

Group B: legs whose volume increased compared with that in the initial visit during complex decongestive therapy.

a *P* < .05 vs initial visit.

**Fig 1:**
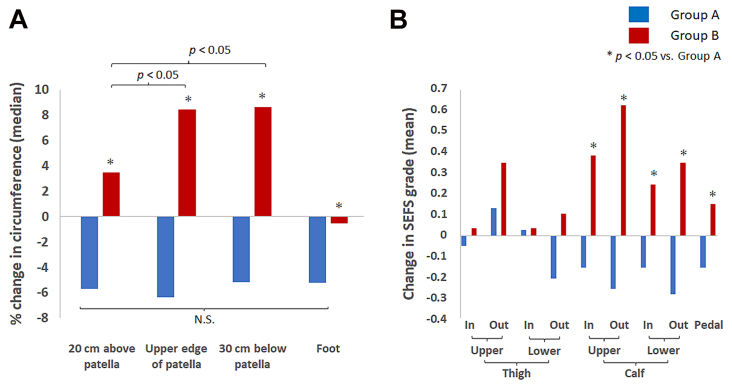
The changes in leg circumferences and subcutaneous echo-free space (*SEFS*) grades during complex decongestive therapy. **A,** Percentage changes in leg circumferences. **B,** Change in mean SEFS grades. Group A: legs that maintained decreased volume during complex decongestive therapy. Group B: legs whose volume increased compared with that in the initial visit during complex decongestive therapy.

In patients with bilateral LLL (n = 16), when one leg grew bigger, the other leg also grew bigger (not necessarily a corresponding equal change) but never became smaller. The overall change in the volume of the affected limbs (n = 68) was –167 mL (–2882 to +3257 mL) during the study period. Decreased limb volume was maintained in 37 limbs (group A, 54%), whereas limb volume increased in 31 limbs (group B, 46%). At the initial visit, the limb volumes between groups A (7522 mL [5297-12,690 mL]) and B (7126 mL [5090-10,890 mL]) were not significantly different (*P* = .37). The change in limb volume in group A was –973 mL (–2881 to –69 mL), whereas the change in group B was +1208 mL (+51 to +3257 mL); each was significant compared with the initial limb volume (*P* < .0001). In group A, the circumference decreased evenly at all assessed levels by approximately 6%. In group B, the circumferences increased by 3% at 20 cm above the patella (upper thigh), 8% at the upper edge of the patella (lower thigh), and 7% at 30 cm below the patella (calf), but not in the foot (Fig 1, *A*). Regarding the condition of the subcutaneous tissue, SEFS decreased in the lower thigh and calf in group A, whereas SEFS increased particularly in the calf in group B (Fig 1, *B*).

### Changes in adherence to compression therapy and its impact on limb volume

Because compression therapy affects limb volume the most among the CDT components, we evaluated changes in adherence to compression therapy. Because adherence may change over a long period of CDT due to patients' conditions, such as age, lifestyle, and physical condition, we reviewed the average adherence in the initial and latest 3 years. The results are summarized in Table II. The frequency of compression stockings and/or bandage use did not significantly change. In contrast, the overall status of compression levels, that is, interface pressures (IP) of the compression devices, was significantly changed. Namely, the use of compression devices exerting lower IPs increased.

**Table II:** Changes in adherence to compression therapy

	Initial 3 years	Latest 3 years	Overall change in adherence status (*P* value)	Changes in group A/B ratio (*P* value)
Frequency of compression therapy	n = 52	n = 52	.22	
≥5 days a week, No. (%)	45 (87)	38 (73)		.80
Group A	26	23		
Group B	19	15		
1-4 days a week, No. (%)	4 (7)	9 (17)		.76
Group A	1	3		
Group B	3	6		
0 days a week, No. (%)	3 (6)	5 (10)		.85
Group A	1	2		
Group B	2	3		
Compression levels	n = 49	n = 47	<.05	
>30 mm Hg, No. (%)	34 (69)	26 (55)		.50
Group A	20	13		
Group B	14	13		
20-30 mm Hg, No. (%)	15 (31)	16 (34)		.10
Group A	7	12		
Group B	8	4		
<20 mm Hg, No. (%)	0 (0)	5 (11)		Not calculated
Group A	0	1		
Group B	0	4		

Group A, B: as in Table I.

**Fig 2:**
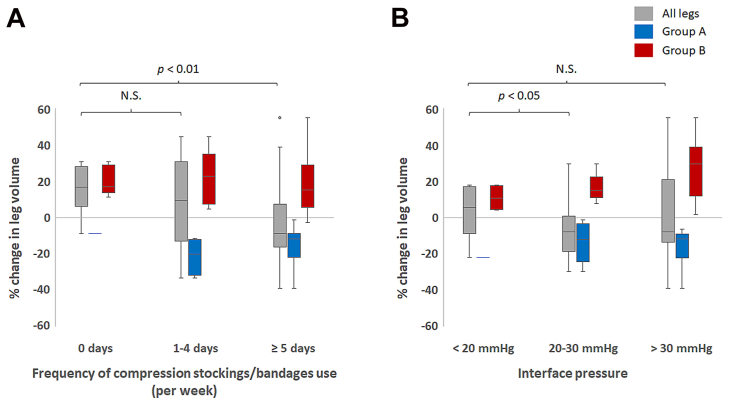
The changes in leg volumes according to adherence status. **A,** Percentage changes in leg volumes according to the frequency of compression device use. **B,** Percentage changes in leg volumes according to the compression level. Group A, B: as in Fig 1.

Changes in limb volume according to the latest adherence to compression therapy are shown in Fig 2. In overall patients, as expected, the limb volume increased compared with that at the initial visit in nonadherent patients. The patients who used compression stockings for 5 days or more per week maintained a reduced limb volume. Patients who used compression stockings for 1 to 4 days per week could not maintain a reduced limb volume, and the change in limb volume was not significantly different from that in nonadherent patients. In each group, the changes in limb volume did not reach significance according to the frequency of the compression device use (Fig 2, *A*).

Regarding the IP of the compression devices, there was a significant difference between IP <20 mm Hg and IP of 20 to 30 mm Hg. The changes in leg volumes treated by the devices with IP >30 mm Hg were largely varied (Fig 2, *B*).

### The change in BMI and its effect on limb volume

The change in BMI was significantly larger in group B than in group A (group A: 0.0 kg/m^2^ [–6.2 to +2.1 kg/m^2^], group B: +0.1 kg/m^2^ [–4.5 to +4.0 kg/m^2^], *P* < .01).

**Fig 3:**
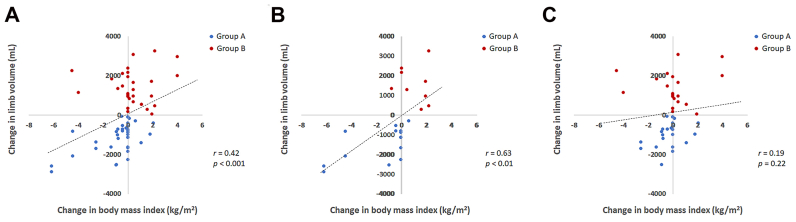
The correlation between the changes in limb volume and body mass index. **A,** Overall patients. **B,** Patients with initial body mass index ≥25 kg/m^2^. **C,** Patients with initial body mass index <25 kg/m^2^. Group A, B: as in Fig 1.

The correlation between changes in BMI and limb volume is shown in Fig 3. Overall changes in BMI and limb volume were weakly correlated (*r* = 0.42, Fig 3, *A*). When the correlation was separately assessed in the legs of the patients with the initial BMI ≥25 kg/m^2^ (Fig 3, *B*) and those with the initial BMI <25 kg/m^2^ (Fig 3, *C*), the former showed a good correlation (*r* = 0.63), whereas the latter did not show a significant correlation using the linear regression analyses.

### Factors related to the increase in limb volume

The factors associated with limb volume increase are summarized in Table III. Among patient-related factors, an increase in BMI was found to be a significant factor in univariate analysis. Among the CDT-related factors, adherence to compression therapy and compression levels were significant in univariate analysis. In multivariate analysis, an increase in BMI, the use of a compression device for fewer than 5 days per week, and IP less than 20 mm Hg were found to be associated with an increase in leg volume during prolonged CDT.

**Table III:** Factors related to the increase in limb volume

	Univariate		Multivariate	
	Odds ratio (95% CI)	*P* value	Odds ratio (95% CI)	*P* value
Patient-related factors				
Latest age	1.01 (0.97-1.05)	.67		
Sex (female vs male)	4.00 (0.48-83.32)	.21		
Side (left vs right)	1.18 (0.45-3.11)	.74		
Latest ISL stage (III vs II)	1.09 (0.22-5.94)	.91		
Increase in body mass index	1.53 (1.11-2.34)	<.01	1.73 (1.15-2.93)	<.01
CDT-related factors				
Duration of CDT	1.15 (0.96-1.39)	.13		
Latest adherence to compression therapy				
Noncompliant vs 1-4 per week	1.13 (0.12-8.78)	.91		
1-4 per week vs ≥ 5 per week	4.10 (1.15-17.04)	<.05	4.57 (1.06-23.53)	<.05
Latest compression level				
<20 mm Hg vs 20-30 mm Hg	19.0 (2.39-411.89)	<.01	36.47 (3.42-1061.9)	<.01
20-30 mm Hg vs > 30 mm Hg	0.25 (0.07-0.79)	<.05	0.46 (0.12-1.73)	.25

*CDT,* Complex decongestive therapy; *CI,* confidence interval; *ISL,* International Society of Lymphology.

Group A, B: as in Table I.

## Discussion

The main findings of this study are as follows: during median 11.2 years of CDT, (1) limb volume increased in 46% of the affected limbs with LLL compared with that at the initial visit; (2) limb volume increase was associated with increased edema, mainly in the calf area; (3) the possible factors contributing to the increase in limb volume were the increase in BMI, the use of compression devices <5 days a week, and IP <20 mm Hg.

### Adherence to compression therapy as a part of CDT

For successful CDT, various factors, including patient and disease characteristics, types of interventions, and adherence to the treatment, are important.^10^ Of those, compression therapy is the most important for maintaining the reduced limb volume obtained in the first phase. At the same time, difficulty in maintaining adherence to compression therapy has been persistently reported.^11^, ^12^, ^13^ The reasons for this poor adherence are mainly physical and economic burdens, but the lack of literacy and psychosocial problems have also been noted.^14^^,^^15^ To overcome these problems, it is helpful to inform patients about what can be achieved by prolonged CDT, and the current results may be quite helpful for this purpose.

The higher IP generally results in more edema reduction.^16^ In this study, however, the use of compression devices with IP >30 mm Hg was not associated with a significantly larger leg volume reduction compared with those with lower IPs. This should not be interpreted as these compression levels exerting similar effects, because their use depended on LLL severity. Moreover, the increase in leg volume is caused not only by the increase in edema but also by adipose tissue proliferation, which is not managed using compression therapy.

Problems with adherence, including both frequency and IP, are considered to be at least partially related to aging. In other words, providing proper CDT for a long period may require the change of strategy to adjust for aging.

### Causes of the increase in leg volume during prolonged CDT

In this study, an increase in limb volume was associated with increased calf edema. In our previous report, which reviewed a median follow-up of 3 years of CDT, we found that the volume increased in 35% of limbs with LLL.^6^ However, this increase was due to an increase in subcutaneous thickness, particularly in the lower medial thigh, possibly caused by exacerbated edema and/or adipose tissue proliferation in this area, of which the mechanism is still unclear, but involvement of impaired reverse cholesterol transport^17^ or upregulation of proteins such as peroxisome proliferator-activated receptor γ^18^ is suggested. This discrepancy in the modes of the increase in limb volume may be related to aging. During prolonged CDT, the median age of the patients increased from 65 to 75 years in this study. Aging is associated with increased vascular permeability,^19^ reduced cardiopulmonary reserve,^20^ reduced skin viscoelasticity,^21^ and reduced lymph transport,^22^ all of which can contribute to edema in dependent positions.

### Effect of BMI on limb volume in patients with LLL

Morbid obesity can cause lymphedema by reducing lymph transport^23^ and can also cause chronic venous insufficiency through mechanical factors (increased intra-abdominal pressure) and inflammatory processes.^24^ Furthermore, obesity can increase lymphatic volume load due to the increased intravascular volume, which would further exacerbate peripheral edema.^25^ Because these conditions occur simultaneously, not independently, in morbidly obese patients, this type of leg edema of complex pathology should be regarded as “obesity-related edema” rather than being considered as one of the patient characteristics. Greene et al^23^ suggested that a threshold for presenting abnormal lymphatic function, that is lymphedema, might exist between 50 kg/m^2^ and 60 kg/m^2^. In this study, the percentage of patients with BMI ≥30 kg/m^2^ was 7.5%, and no patients had a BMI >40 kg/m^2^. This means that the patients included in the current study were least likely to be complicated by obesity-related lymphatic insufficiency.

In the lower limbs without LLL, there was a good correlation between limb volume obtained from tape measures and BMI.^26^ Although limbs with LLL were affected by edema and pathological adipose tissue deposition, a weak correlation still existed between the changes in limb volume and BMI. This indicates that weight loss can have a beneficial effect on reducing the volume of limbs with LLL. It has been reported that weight reduction can contribute to the reduction of adipose tissue, particularly in the thigh area,^27^ which is difficult to achieve with compression therapy alone. Therefore, weight reduction may be recommended not only to reduce edema but also to reduce adipose tissue. This also indicates that other approaches to reduce body weight, for example, glucagon-like peptide-1, may be options to manage LLL.

### Limitations

This study had some limitations that should be considered. First, it was conducted at a single center with a limited patient population, which could limit the generalizability of the findings. This resulted in unusual odds ratios and wide 95% confidence intervals. Second, the assessment of adherence may have been inappropriate. We only assessed the initial and latest 3 years, but the adherence between these periods might have affected the limb volume. In addition, we could not capture the use of nonmedical compression devices at home, which could also have affected the results. Third, we focused on the limb volume in this study. However, controlling limb volume is not the sole purpose of CDT. Rather, other goals, such as maintaining physical function, reducing psychological distress, and preventing complications, may be even more important in managing noncurable diseases. Therefore, future studies should assess the long-term effects of CDT from multiple perspectives.

## Conclusions

During prolonged CDT, limb volume increased in 46% of limbs with LLL, primarily due to increased edema, particularly in the calf area. Factors associated with this increase included the increase in BMI, use of compression devices <5 days a week, and IP <20 mm Hg. Therefore, compression needs to be better tailored to patients as they age, and strict weight control is crucial to manage LLL during prolonged CDT.

## Author contributions

Conception and design: KS

Analysis and interpretation: KS, TH, YT, TM, HK, MS, KH

Data collection: KS, TH, YT, TM, HK

Writing the article: KS

Critical revision of the article: KS, TH, YT, TM, HK, MS, KH

Final approval of the article: KS, TH, YT, TM, HK, MS, KH

Statistical analysis: KS, MS

Obtained funding: Not applicable

Overall responsibility: KS

## Funding

None.

## Disclosures

None.
